# A Novel Methodology to Evaluate Health Impacts Caused by VOC Exposures Using Real-Time VOC and Holter Monitors

**DOI:** 10.3390/ijerph7124127

**Published:** 2010-11-30

**Authors:** Atsushi Mizukoshi, Kazukiyo Kumagai, Naomichi Yamamoto, Miyuki Noguchi, Kazuhiro Yoshiuchi, Hiroaki Kumano, Yukio Yanagisawa

**Affiliations:** 1 Tokyo Metropolitan Industrial Technology Research Institute, Nishigaoka, 3-13-10, Kita-ku, Tokyo 115-8586, Japan; 2 Department of Environment Systems, Graduate School of Frontier Sciences, The University of Tokyo, Kashiwa-no-ha 5-1-5, Kashiwa-shi, Chiba 277-8563, Japan; E-Mails: miyuki_noguchi@yy.k.u-tokyo.ac.jp (M.N.); yukio@k.u-tokyo.ac.jp (Y.Y.); 3 Environmental Health Laboratory Branch, California Department of Public Health, 850 Marina Bay Pkwy, Richmond, CA 94804, USA; E-Mail: kazukiyo.kumagai@cdph.ca.gov; 4 Research and Education Center of Carbon Resources, Kyushu University, 6-1, Kasugakoen, Kasuga, Fukuoka 816-8580, Japan; 5 Environmental Engineering Program, Yale University, 9 Hillhouse Ave., New Haven, CT 06520, USA; E-Mail: naomichi.yamamoto@yale.edu; 6 Japan Society for the Promotion of Science (JSPS), Ichiban-cho 8, Chiyoda-ku, Tokyo 102-8472, Japan; 7 Department of Psychosomatic Medicine, Graduate School of Medicine, The University of Tokyo, Hongo 7-3-1, Bunkyo-ku, Tokyo 113-8655, Japan; E-Mail: kyoshiuc-tky@umin.ac.jp; 8 Faculty of Human Sciences, Waseda University, Mikashima 2-579-15, Tokorozawa-shi, Saitama 359-1192, Japan; E-Mail: hikumano@waseda.jp (H.K.)

**Keywords:** real-time monitoring, VOCs, heart rate variability (HRV), Holter monitor

## Abstract

While various volatile organic compounds (VOCs) are known to show neurotoxic effects, the detailed mechanisms of the action of VOCs on the autonomic nervous system are not fully understood, partially because objective and quantitative measures to indicate neural abnormalities are still under development. Nevertheless, heart rate variability (HRV) has been recently proposed as an indicative measure of the autonomic effects. In this study, we used HRV as an indicative measure of the autonomic effrects to relate their values to the personal concentrations of VOCs measured by a real-time VOC monitor. The measurements were conducted for 24 hours on seven healthy subjects under usual daily life conditions. The results showed HF powers were significantly decreased for six subjects when the changes of total volatile organic compound (TVOC) concentrations were large, indicating a suppression of parasympathetic nervous activity induced by the exposure to VOCs. The present study indicated these real-time monitoring was useful to characterize the trends of VOC exposures and their effects on autonomic nervous system.

## 1. Introduction

Volatile organic compounds (VOCs) are of great concern because of their potential wide-ranging health impacts [1]. For instance, most VOCs observed in buildings are known to cause neurological toxicity [2] and clinical symptoms such as headaches, nausea, respiratory irritation, fatigue, complaints, asthma symptoms and so on [3,4]. Furthermore, some VOCs are suggested to be related to sick building syndrome (SBS) and/or multiple chemical sensitivity (MCS) and induce the above-mentioned symptoms even by exposures to the small amounts present in general environments [4–7]. Nevertheless, the detailed mechanisms of these effects have not been clarified yet. To elucidate these mechanisms, simultaneous measurements of both VOC exposures and health impacts are essential.

In general, to assess personal exposures to VOCs, the exposure concentrations are inevitably averaged over several hours or days since it takes time to collect the sufficient amounts of VOCs necessary for subsequent analytical procedures such as gas chromatography-mass spectroscopy (GC-MS) analysis. However, since the personal exposures are fluctuate considerably due to personal activities as well as the microenvironments where the individuals are present [8,9], time-resolved measurements are often essential to accurately characterize the personal exposure patterns. Several time-resolved methods including proton transfer reaction mass spectrometry (PTR-MS) and selected ion flow tube-mass spectrometry (SIFT-MS) have been developed [10–13]. However, these methods cannot be used for personal exposure measurements because of the size of the instruments.

A recently developed portable real-time monitor to measure VOCs could be a useful tool to overcome these difficulties [14,15]. Among the various kinds of portable VOC monitors, the monitor using a photoionization detector (PID) can detect total VOCs (TVOCs) at intervals of a few seconds, and the detection sensitivity by this detector was reported to range from ppb to ppm [16]. Furthermore, a wide range of VOC species can be detected by PID. For instance, in the cases of a 10.6-eV UV lamp, more than one hundred VOC species including benzene, trichloroethylene, tetrachloroethylene, toluene, xylene, *p*-dichlorobenzene, ethylbenzene, styrene and so on are detectable as TVOCs. Therefore, the PID-based VOC monitor is expected to be a good tool to accurately measure time-resolved personal VOC exposure data for human subjects.

Meanwhile, heart rate variability (HRV), which reflects the function of an autonomic nervous system, is often measured to evaluate health impacts caused by various environmental factors. Since the heart rates are regulated by an autonomic nervous system, the measurement of HRV can directly indicate the status of the autonomic nervous system. By analyzing the frequency domains of the RR intervals (that is, intervals between adjacent R waves resulting from sinus node depolarizations), typical spectral peaks appear in the ranges of low frequency (LF, 0.04–0.15 Hz) and high frequency (HF, 0.15–0.4 Hz) in the power spectrum density [17]. While HF power mainly reflects parasympathetic activity, LF power primarily reflects sympathetic activity with some parasympathetic input. The relative power of LF to HF components (that is, power ratio of LF to HF) is often used as an indicator of sympathetic nervous activity, too [17,18].

To date, numerous studies have been conducted to investigate the relationships between HRV and diseases and/or clinical symptoms [19–22]. Since a lightweight portable electrocardiogram (ECG) Holter monitor has become available recently, HRV has been widely measured for individuals along with measurements of various environmental factors such as airborne particulate matter [23–28], carbon oxide [26], environmental tobacco smoke [29], electromagnetic fields and so on [30]. To our knowledge, however, no study has ever been conducted to measure HRV along with personal VOC exposures.

The present study aims to report a methodology to evaluate health impacts caused by personal exposures to VOCs by using the abovementioned VOC and ECG monitors. We used the PID-based VOC monitor and the ECG Holter monitor to measure personal TVOC exposures and HRV, respectively. Since these monitors are portable and capable of obtaining time-resolved data, the real-time simultaneous monitoring of both personal VOC exposures and HRV becomes possible. In this study, we analyzed the time-series data of the personal VOC exposure and HRV of seven normal individuals and examined the relationship between these two variables.

## 2. Method

### 2.1. Study Design

This study was designed to simultaneously monitor personal VOC concentrations and HRV for seven normal subjects under usual daily life conditions. The measurements were conducted from December 2006 to January 2007. The subjects were requested to wear the Holter monitor, carry the VOC monitor and record time-activity logs during monitoring. This study was approved by the Human Ethical Committee of the Kitasato Institute Hospital.

### 2.2. Subjects

In this study, seven human subjects including four adult males and three adult females were asked to participate in our monitoring campaigns. The procedure was explained and informed consent was obtained from all the subjects. Among them, six subjects were recruited from the members of the laboratory of the University of Tokyo and the remaining one subject was recruited by public announcement. They were normal individuals with no previous record of diagnosed SBS or MCS. The ages of the subjects ranged from 22 to 54 years (32 ± 13 years).

### 2.3. HRV Analysis

The continuous electrocardiogram (ECG) data were recorded for 24 hours by the Holter monitor [FM-150, 49.5(W) × 44.5(H) × 14.7(D) mm, 40 g, FUKUDA DENSHI, Japan]. The subjects were instructed how to attach four electrodes in proper positions: in the upper segment of the sternum as ch1/2 (−), the lower segment of the sternum as ch2 (+), the V5 position as ch1 (+) and theV5R position as reference. The Holter monitor was worn around his/her necks and was fixed on a lower abdomen with a seal to avoid data noise. The subjects were asked to go about their normal daily life activities during the measurement period, but without taking a bath to prevent the failure of the equipment. The ECG data were collected at 125 Hz and recorded on removable multimedia cards.

In the analysis of HRV, RR intervals of ECG were used. According to Task Force, 250-Hz is recommended as sampling rate, while a lower sampling rate may behave satisfactorily only if an algorithm of interpolation is used [17]. In this study, cubic spline interpolation was conducted to RR curve of ECG. After interpolation, HF and LF power were calculated in each 10-sec interval by the Gabor wavelet transform, which is a new methodology for frequency analysis in quantifying HRV in non-stationary conditions [31]. The Cubic spline interpolation and the Gabor wavelet transform analysis was conducted using Fluclet WT (Dainippon Sumitomo Pharma, Japan), a software implementing the Gabor transform especially for circulatory dynamics parameters. The calculated HF and LF power were averaged over 5-min interval according to the recommendation of Task Force [17]. As mentioned before, HF power was used as an indicator of parasympathetic activity and the power ratio of LF to HF (LF/HF) was used as an indicator of sympathetic activity.

### 2.4. TVOC Monitoring

The exposure concentrations of TVOCs were measured for 24 hours by a portable real-time VOC monitor with PID [ppbRAE, 76.2 (W) × 50.8 (H) × 218 (D) mm, 553 g, RAE systems, USA)]. The detector was equipped with 10.6-eV UV lamp. Before each measurement, calibrations were conducted using VOC-free air (pure air, G1) and 10 ppm isobutylene gas. To prevent possible psychological biases caused by perceiving the monitored values of the VOC monitor by the subjects, the subjects were blinded for the reading of the monitor by sealing on the display. In addition, to reduce the noise of the pump during the measurements, the monitor was put in a soundproof box filled with cotton. The subjects were requested to put the monitor into a bag and carry and/or keep it aside.

The monitor can measure TVOC concentrations every second, but since the data logging capacity is limited, the averaged concentrations of 20 sec or 1 min intervals were recorded during the 24-hour measurement periods. At the same time, the monitor was configured to record the maximum concentrations and minimum concentrations for each interval. The recorded values were isobutylene equivalent concentration in ppb. Isobutylene is used to calibrate PIDs because its responsiveness by PID is relatively moderate among the VOCs generally observed in the general environment. Furthermore, it is easy to handle since it is non-toxic and non-flammable at the low concentration used for calibration [16]. However, since isobutylene is not a major component in the general environment, it is unrealistic to denote TVOC concentrations as isobutylene equivalent concentrations. Instead, we converted isobutylene equivalent concentrations to toluene equivalent concentrations, a commonly-used expression for TVOCs, using a correction factor. Correction factor is used to adjust the sensitivity of PID to convert isobutylene equivalent concentrations to toluene equivalent concentrations of measured gaseous substances. Toluene equivalent concentration is calculated using the follow equation:

(1) $$C_{toluene}=C_{isobutylene}\times {CF}_{toluene}$$

where C*_toluene_* [ppb] is toluene equivalent concentration of TVOCs, C*_isobutylene_* [ppb] is isobutylene equivalent concentration of TVOCs and CF*_toluene_* is the correction factor for toluene (=0.5 [16]). Moreover, C*_toluene_* was converted from ppb to μg/m^3^ with the temperature adjustment. Temperatures were measured by thermohygrometer (HOBO, Onset Computer Corporation, USA). These values were averaged over each 5-min interval. Relative humidity (RH) was also measured by the thermohygrometer. In addition to analyzing the absolute concentrations of TVOCs, we analyzed the changes of TVOC concentrations within a certain interval of measurement period. In this study, the changes of TVOC concentrations for each 5-min interval, expressed as ΔTVOC (μg/m^3^), were defined as the difference of the minimum and maximum concentrations measured in that 5-min interval. ΔTVOC, which is an absolute value of the concentration difference, cannot distinguish whether it was increased or decreased between the consecutive time intervals. Here we defined d+TVOC and d−TVOC if the difference was positive and negative, respectively.

### 2.5. Time-Activity Pattern

**Figure 1:**
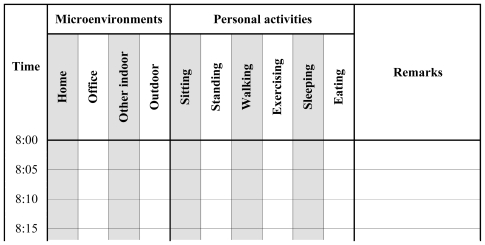
An excerpt of a time-activity log sheet.

The time-activity patterns in each 5-min interval were recorded by the subjects. The subjects were requested to select their activities and environments from the following alternatives shown in Figure 1; six kinds of personal activities including sitting, standing, walking, exercising, eating and sleeping and 4 kinds of microenvironments including home, office, other indoor and outdoor.

## 3. Results and Discussion

### 3.1. Time Series Data

**Figure 2:**
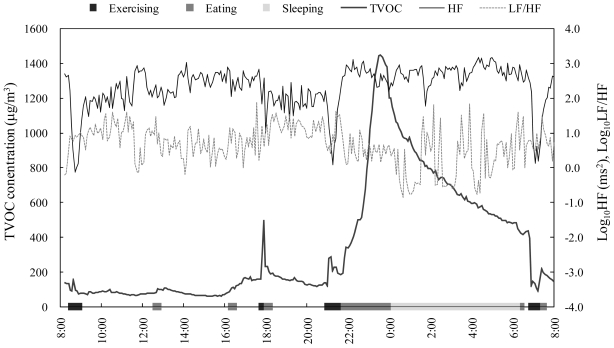
An example of time-series data of TVOC concentration and HRV (Subject a).

Figure 2 shows an example of the time series data. TVOC concentrations varied notably in this measurement period (mean ± SD, 354 ± 349, range 63–1,447 μg/m^3^) and several TVOC peak concentrations were observed. The peaks observed at 8:40 and 21:05 were suspected to be of outdoor origin because the subject was outdoors according to the time-activity log. Meanwhile, the peaks observed between 22:00 and 24:00 were likely due to cooking activities. Unlike the traditional time-integrated measurements, the time-resolved measurements can provide considerable information regarding personal VOC exposures. Furthermore, using with the time-resolved time-activity data, the trends of the personal exposures can be accurately characterized and the linkages between the time activities and the TVOC concentrations can be effectively assessed.

In Figure 2, HRV parameters also fluctuated through the measurement period. In particular, Figure 2 indicates that the HRV parameters were changed by the personal activities. For instance, HF was decreased during exercising and eating and increased during sleeping. To date, the relationships between HRV and physiological conditions have been extensively investigated [32–34]. For example, absolute values of HF are known to decrease with exercising [32]. In addition, LF/HF and HF were increased and decreased, respectively, for 1 hour after eating, suggesting the physiological conditions such as increased heart rate, blood pressure, and cardiac output commonly observed after food ingestion could affect HRV [33]. Moreover, it was revealed LF was decreased during sleeping, while HF was increased with sleep onset [34]. Thus, HRV was significantly affected by physiological conditions. Therefore, to ignore these confounding factors, we excluded the data of the time spent for these activities (*i.e.*, exercising, eating and sleeping) and the time the effect continue (*i.e.*, 15 min after exercising, 1 hour after eating) from the analyses.

### 3.2. Statistical Summary

Table 1 shows a summary of the exposure concentrations of TVOCs and HRV parameters observed for all subjects studied in this study. The concentrations of TVOCs measured are in the ranges normally observed in exhaled breath [35–37]. The results indicate the exposure concentrations differed by each microenvironment. The results also indicate the inter-individual variation in the exposure concentrations at office was relatively small probably because 6 subjects stayed in the offices of the same building.

### 3.3. Bivariate Analysis

Spearman rank correlation coefficients were calculated by using JMP7 (SAS) to assess the relationships between TVOC exposures and HRV parameters measured within the same 5-min intervals. Table 2 shows a summary of the correlations for all subjects. The sex and age of these subjects are listed in Table 2. The significant negative correlations between ΔTVOC and HF were observed in six out of seven subjects, suggesting the changes of TVOC concentrations in 5-min were associated with the decrease of the activity of parasympathetic nervous. Meanwhile, one out of seven subjects showed significant negative correlations between TVOC concentrations and HF. Based on these results, the HF decreases were more likely due to the changes of TVOC concentrations rather than the absolute values of TVOC concentrations. The significant positive correlations were observed between ΔTVOC and LF/HF in five subjects, too, suggesting the changes of TVOC concentrations in 5-min were associated with the enhancement of the activity of the sympathetic nervous. Despite the difference of sex and age, these tendencies, negative correlation with ΔTVOC and HF and positive correlation of ΔTVOC and LF/HF, were observed excluding Subject f, whose correlation of ΔTVOC and LF/HF was negative. As a result, ΔTVOC could be a potential candidate to evaluate the influence of VOC exposures on the autonomic nervous system.

At the same time, the tendency of negative correlation between d+TVOC and HF (seven subjects) and the tendency of positive correlation between d−TVOC and HF (seven subjects) were observed. Moreover, the tendency of positive correlation between d+TVOC and LF/HF (five subjects) and the tendency of negative correlation between d−TVOC and LF/HF (five subjects) were observed. These tendencies suggested that TVOC concentration increases and decreases promote different effects on the successive HRV parameters. In contrast, the consistent tendencies were not observed between temperature and RH and HRV.

For other pollutants, decreases of HRV parameters with increased pollutant concentration were often observed [23–29]. In the case of submicrometer particles (size range 0.02–1 μm), both immediate effects such as direct myocardial effects and cumulative effects such as increase blood coagulation by deposition of ultrafine particles in the alveoli were suggested [24]. The mechanism of TVOC effect on nerve system is not clear, but pollutants including TVOCs, which are classified as environmental stressor, may generally change of HRV.

Since these correlations might also be affected by other unknown confounding factors such as psychological stress, climate conditions, other environmental factors and so on, future studies will be necessary to further elucidate the underlying causal mechanisms between these two variables. However, our findings suggest the possibility of the some kind of reaction to VOCs of human subjects, even normal subjects who experience no subjective symptoms.

Since different VOC species may have different degree of impacts on HRV, using TVOCs to study the potential autonomic defect of VOC exposure has its limitations. For example, because the compositions of VOCs might differ in each microenvironment, the health effects might also differ. To adjust for such differences, the correlations between TVOC exposures and HRV parameters were investigated for each microenvironment (*i.e.*, home, office, other indoor and outdoor). Table 3 shows the ranges and medians of the correlation coefficients and the numbers of the subjects showing significant correlations between TVOC exposure and HRV by each environment (Spearman rank correlation coefficients, p < 0.05). In indoor environments such as home, office and other indoors locations, the ranges of the correlation coefficients between ΔTVOC and HF were smaller than those between TVOC concentration and HF and the median values were negative. In the case of the correlation coefficients between ΔTVOC and LF/HF, the ranges were not always smaller than those between TVOC and LF/HF, but the median values were positive. Moreover, the significant negative correlations between ΔTVOC and HF were observed in 2–4 subjects in indoor environments. Thus, the correlations between ΔTVOC and HRV parameters observed indoors were more distinctive than those observed outdoors. Although the underlying mechanisms remain to be unknown, the present data suggest indoor VOCs had more influence on the autonomic nervous system than outdoor VOCs.

## 4. Conclusions

In this study, real-time measurements of personal VOC exposure and HRV under usual daily life conditions were conducted for several human subjects. The results indicated significant correlations between ΔTVOC and HRV parameters. The methodology reported here, which is relatively noninvasive and easy to carry out in the field surveys, can be used to evaluate the relationships between VOC exposures and autonomic function continuously under usual daily life conditions. The present methodology could be used in the future to evaluate the health impacts caused by VOC exposures of MCS patients.

## Figures and Tables

**Table 1:** Summary of TVOC exposure concentrations and HRV parameters.

	
	n a	Mean ± SD b	CV (%) c
TVOC exposure concentration (μg/m^3^)			
Total	7	176 ± 130	74
Home	7	299 ± 267	89
Office	6	99 ± 21	21
Other indoor	5	197 ± 118	60
Outdoor	6	154 ± 72	47
HRV			
HF (msec^2^)	7	104.2 ± 89.0	85
LF/HF	7	15.3 ± 19.0	124

a Sample size

b Standard deviation

c Coefficient of variance

**Table 2:** Correlation between TVOC exposure and HRV.

	Subjects							−c	+^d^
	a	b	c	d	e	f	g		

Sex (M: male, F: female)	M	M	F	M	F	M	F		
Age (years)	24	22	22	23	48	33	54		
TVOC *vs* HF	−0.41**^a^	−0.04	−0.00	0.02	−0.15	−0.01	−0.08	6(1)	1(0)
ΔTVOC *vs* HF	−0.27**	−0.63**	−0.26**	−0.08	−0.19*^b^	−0.32**	−0.26 **	7(6)	0(0)
d+TVOC *vs* HF	−0.11	−0.53**	−0.15	−0.06	−0.04	−0.12	−0.43 **	7(2)	0(0)
d−TVOC *vs* HF	0.41**	0.66**	0.18	0.16	0.29*	0.23	0.33 **	0(0)	7(4)
TVOC *vs* LF/HF	0.37**	0.05	−0.16*	0.15	0.10	0.01	−0.17 *	2(2)	5(1)
ΔTVOC *vs* LF/HF	0.20*^b^	0.39**	0.07	0.18*	0.18*	−0.27**	0.36 **	1(1)	6(5)
d+TVOC *vs* LF/HF	0.15	0.28*	−0.00	0.24*	0.14	−0.18	0.46 **	2(0)	5(3)
d−TVOC *vs* LF/HF	−0.30*	−0.46**	0.01	−0.18	−0.07	0.13	−0.45 **	5(3)	2(0)
Temperature *vs* HF	0.64**	−0.14	−0.15	0.07	−0.20	0.10	0.14	3(0)	4(1)
RH *vs* HF	−0.51**	0.11	−0.08	−0.00	0.29	−0.00	0.10	4(1)	3(0)
Temperature *vs* LF/HF	−0.44**	0.14	0.17*	−0.05	−0.00	0.30**	−0.25 **	4(2)	3(2)
RH *vs* LF/HF	0.31**	−0.07	−0.01	0.07	0.00	−0.21**	−0.35 **	4(2)	3(1)

a Spearman rank correlation, p < 0.01

b Spearman rank correlation, p < 0.05

c Numbers of the subjects showing negative correlation (significant)

d Numbers of the subjects showing positive correlation (significant)

**Table 3:** Range and median of correlation coefficients and number of the subjects showing significant correlation between TVOC exposure and HRV (Spearman rank correlation, p < 0.05).

	Home (n = 7)				Office (n = 6)				Other indoor (n = 5)				Outdoor (n = 4)			
	Range	Median	− a	+ b	Range	Median	−	+	Range	Median	−	+	Range	Median	−	+
TVOC *vs* HF	0.98	0.22	0	1	0.85	−0.28	4	0	1.11	0.19	0	0	0.63	0.17	0	0
ΔTVOC *vs* HF	0.96	−0.24	3	0	0.42	−0.26	4	0	1.04	−0.40	2	0	1.08	0.22	0	0
TVOC *vs* LF/HF	0.54	−0.12	1	0	0.41	0.10	0	2	1.31	0.09	1	0	0.62	−0.03	0	0
ΔTVOC *vs* LF/HF	0.99	0.27	0	1	0.45	0.14	1	2	1.34	0.34	0	2	0.80	−0.05	0	0

a Numbers of the subjects showing negative correlation (significant)

b Numbers of the subjects showing positive correlation (significant)
